# Bilateral pneumothorax: The cause of hypoxia during endoscopic retrograde cholangiopancreatography

**DOI:** 10.22088/cjim.12.0.426

**Published:** 2021

**Authors:** Saeed Madani, Rohallah Taghavi, Mohammad Saiidi, Jamshid Vafaeimanesh

**Affiliations:** 1 Clinical Development Research Center, Qom University of Medical Sciences, Qom, Iran; 2 Qom Gastroenterology and Hepatology Disease Research Center, Qom University of Medical Sciences, Qom, Iran

**Keywords:** Pneumothorax, ERCP, Hypoxemia

## Abstract

**Background:**

Endoscopic retrograde cholangiopancreatography (ERCP) is recognized as a significant diagnostic and therapeutic procedure for the administration of different pancreatic and biliary problems. This procedure runs a considerable risk of complications despite its substantial safety. The rate of significant inconveniences is reported to range from 5.4% to 23.0% and the general mortality from 0.1 to 1%. Post-ERCP pneumothorax is an uncommon complication that is usually underestimated

**Case Presentation:**

In the present study, we report a 65-year-old woman who develops hypoxemia during the ERCP. Based on the obtained results, it was revealed that this patient had perforation-related bilateral pneumothorax and hypoxemia.

**Conclusion:**

Based on the obtained results, it was revealed that this patient had perforation-related bilateral pneumothorax and hypoxemia.

Endoscopic retrograde cholangiopancreatography (ERCP) is recognized as a significant diagnostic and therapeutic procedure. The use of this technique which is commonly performed to diagnose and resolve various pancreatic and biliary tract problems has been reported to be on the rise. In a population-based study conducted in the United States, the average use of ERCP increased from 58 to 105 per 100,000 people per year over 10 years within 1997-2006 (1). This procedure runs a considerable risk of complications despite its substantial safety. In this regard, numerous investigations have been performed on the rate of post-ERCP complications (2). The speciﬁc post-ERCP intricacies reported by studies include pancreatitis, bleeding, sepsis, and perforation. In a synopsis of 21 studies conducted on 16,855 patients within 1987-2003, speciﬁc intricacies were reported as 1154 (6.9 percent) with 55 deaths (0.33 percent) (3). In another study, the rate of significant entanglements went from 5.4% to 0.23% and the general mortality from 0.1 to 1% (4, 5). Moreover, the negative side effects of general anesthesia are other post-ERCP complications since this procedure is performed with anesthesia in many centers. Also, sedation-induced hypoxemia is commonly observed in endoscopic procedures. In the case of upper endoscopy, oxygen desaturation is most commonly attributed to the use of sedation rather than the passage of an endoscope along the airway (6). Some degree of hypoxemia is inevitable during endoscopy, even in patients who do not receive procedural sedation (7). Patients who undergo ERCP are more susceptible to hypoxemia due to their special conditions. However, it is worthy to note that the cause of hypoxemia is not always sedative.

One of the rare complications during ERCP is pneumothorax, which occurs due to retroperitoneal rupture of the duodenum (8). Duodenal perforation complicating ERCP is phenomenal. It is assessed to be somewhere in the range of 0.1 and 1% (9-10) having a mortality of 16–33.3% (10-11). A sensational intricacy of retroperitoneal perforation is the advancement of pneumothorax. As this is an uncommon, sudden, startling and conceivably hazardous function, each one of those engaged with the consideration of patients going through ERCP ought to know about this expected intricacy and know about the etiology, restorative standards and anticipation. The occurrence of this complication can cause fatal hypoxia in the patient.

In the present article, we report the instance of a patient who developed hypoxemia during the ERCP procedure. Also, we found that this patient had perforation-related bilateral pneumothorax and hypoxemia.

## Case Presentation

Our case was a 65-year-old elderly woman with an ongoing scene of jaundice and abdominal pain which was settled with medical therapy. The transabdominal ultrasound was indicative of the presence of stone inside the dilated common bile duct (CBD) and she was alluded to an elective outpatient ERCP owing to choledocholithiasis. The assessment was average; in addition, aspartate aminotransferase, alanine aminotransferase, and bilirubin were seen to be inside the ordinary range. Nonetheless, alkaline phosphatase was elevated (659 IU/dl). Her vital sign was stable, oxygen therapy with nasal cannula was established and oxygen saturation was 98%.The patient underwent general anesthesia with midazolam, fentanyl, and ketamin at the proneposition. 

**Figure 1:**
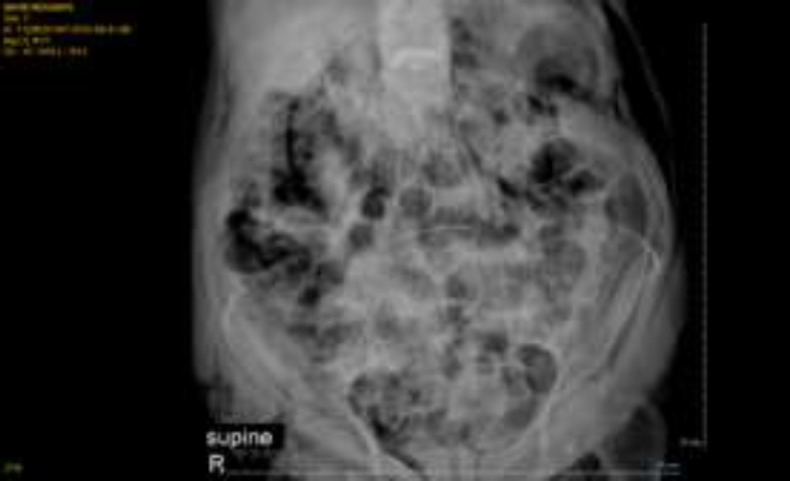
Air inside the abdominal cavity and kidney (retroperitoneal)

**Figure 2:**
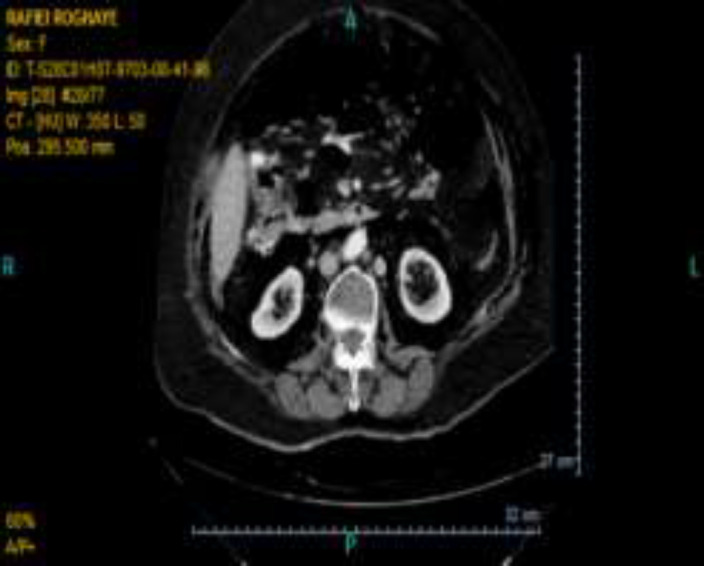
Air inside the abdominal cavity and kidney (retroperitoneal)

**Figure 3:**
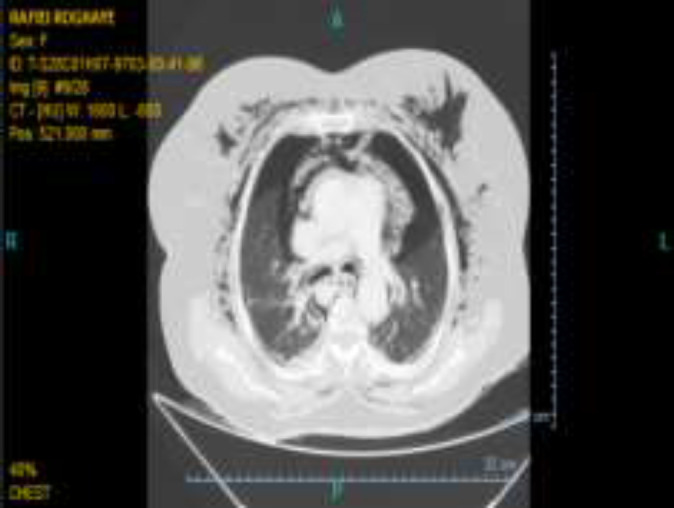
Bilateral pneumothorax and subcutaneous emphysema

The side view endoscope was inserted slowly and easily into the duodenum, and a papilla with a normal appearance was seen next to a small diverticulum anfistolotomy was performed using a kindle knife following technically unsuccessful cannulation of CBD with a standard sphincterotome. Meanwhile, pulse oximetry dropped to 80% and the procedure was interrupted. The patient was released from pronepositin and auxiliary oxygen was applied with bag mask ventilation; nonetheless,the pulse oxymetry did not exceed 82% and there was no obvious reduction in lung sounds at that moment. Also, the patient developed abdominal distension. Therefore, duodenum perforation was suspected and abdominal radiography was performed which disclosed a large area of mottled air in the retroperitoneal area (around the kidney) (figure 1). The physicians made the patient NPO and Iv administration of metronidazole and ceftriaxone was started. Subsequently, facial, cervical, and thoracic subcutaneous emphysema occurred. Oxygen therapy continued with the reservoir bag mask, pulseoxymetry dropped to 75% and bilateral lung sounds decreased. Abdominal computed tomography indicated the evidence of duodenal rupture, including retroperitoneal air, intra-abdominal free fluid, bilateral pneumothorax, Pneumomediastinum and subcutaneous emphysema (figures 2-3).

Bilateral chest tube was inserted instantly and the patient created indications of peritonitis. Laprotomy and repair of a lease (2 cm) situated in the posterolateral mass of the second segment of duodenum (Stapfer type 1 perforation) was performed. At long last, the patient was moved to the ICU, and after 4 days she was moved from the emergency unit to the ordinary ward. She bit by bit improved and was released from the clinic 8 days after the procedure.

## Discussion

Pneumothorax is a rare complication in ERCP (2), but it is extremely life-threatening and can save the patient with prompt diagnosis and treatment. During ERCP, due to the patient's anesthesia and prone position, physicians often associate hypoxia with hypoventilation and airway problems, but this clinical suspicion of the possibility of pneumothorax should be considered (6). Pneumothorax is normally associated with the presence of retroperitoneal, mediastinal and subcutaneous air, oftentimes in addition to intraperitoneal air (10).

ERCP-induced perforationsare reported to be of two typesretroperitoneal and intraperitoneal. Retroperitoneal perforations are usually detected in the periampullary area and arise due to sphincterotomy or guidewire-related perforation. On the other hand, intraperitoneal perforations commonly occur in the lateral wall and endoscopy-related perforation). ERCP-related perforations are classiﬁed by Stapfer et al. into four kinds depending on the seriousness and anatomical area (type I: lateral or medial duodenal wall perforations; type II: peri-vaterian injury; type III: bile or pancreatic duct injury; and type IV: presence of retro- peritoneal air alone (12). 

Nonetheless, perforation needs immediate diagnoses and prompt treatment since deferred diagnosis and management of perforation may prompt the advancement of sepsis and multi-organ failure which causes higher mortality (8% to 23%). (13) symptoms and signs which fuel the suspicion of perforation include epigastric and back pain (more exceptional than expected), subcutaneous emphysema, tachyarrhythmia, tenderness with or without peritoneal signs, and fever (14). Although, tachyarrhythmia is a more constant physical finding, it may not be a reliable indicator of perforation since it can result from other factors, such as pain. Fever and leukocytosis are mostly observed 12 hours or more after the finishing of ERCP. Indications of peritonitis typically take a few hours to create when the duodenal substance are secreted into the peritoneal cavity (15). A critical plain abdominal (AXR) would uncover free extraluminal air, extraluminal retroperitoneal air or contrast (16). The presence of perforation can be detected using computed tomography (CT) of the abdomen and pelvis by oral contrast as the most specific and sensitive symptomatic methodology (17).

 Pneumothorax which is a very uncommon but life-threatening complication can be caused by ERCP-induced retroperitoneal perforation, Post-ERCP pneumothorax is an uncommon complication which is usually underestimated. Supposedly, of our knowledge, very few cases of post-ERCP pneumothorax have been reported in the literature. These cases usually present with bilateral and right-sided pneumothorax which is mostly followed by SCE and pneumomediastinum. The absence of clinical highlights of peritonitis in these patients may pose a diagnostic dilemma. It is noteworthy that the potential risk factors include juxtapapillary diverticula, sphincterotomy, female gender, and older age (>60 years).

Jha AK, detailed two instances of pneumothorax following ERCP and sphincterotomy for choledocholithiasis, one was a 65‐year‐old female. Ultrasonography showed a bile duct stone measured 6mm. After ERCP, the patient created surprising oxygen desaturation following the sphicterotomy. She was noted to create subcutaneous emphysema (SCE) reaching out from the upper chest up to the eyelids. Diminished air section and hyperresonant notes were available on the correct side of chest. Processed tomography (CT) examine exhibited right pneumothorax, pneumomediastinum, and pneumoretroperitoneum. The traditionalist treatment was performed with anti-infection agents, intravenous liquids, chest tube seepage and mechanical ventilation. After two days, SCE began diminishing, pneumothorax relapsed, and the patient was extubated. Another case was A 25‐year‐old lady with cholelithiasis and choledocholithiasis was alluded for ERCP. Cholangiogram showed a stone.The quiet created oxygen desaturation, stomach distension, and SCE not long after the sphicterotomy. CT check demonstrated reciprocal pneumothorax, pneumomediastinum, SCE, pneumoperitoneum, and pneumoretroperitoneum. The understanding created indications of peritonitis. Laprotomy and fix of a lease (2 cm) situated in the posterolateral mass of the second segment of duodenum (Stapfer type 1 hole) was performed. The patient was hence released in sound condition (18). L. Schiavon depicts about a 79-year-old elderly person widened normal hepatic conduit with stone inside and she was alluded to an elective outpatient ERCP due to choledocholitiasis. 

An 8 mm single stone was seen in the common bile duct after a technically difficult sphincterotomy with a standard sphincterotome. When the technique was done, facial, cervical and thoracic subcutaneous emphysema were taken note. A chest x-beam uncovered diffuse subcutaneous emphysema and a right-side pneumothorax. These discoveries were affirmed by a thoracic processed tomography (CT) that additionally showed pneumomediastinum. Stomach CT was mediocre. The patient was made NPO and intravenous organization of prophylactic metronidazole and cephtriaxone was started. The patient was released on emergency clinic day 10 (19). 

Al-Asha reported an instance of strain pneumothorax following an ERCP, which we effectively treated with chest tube addition and laparotomy, and efficiently audit the other 10 cases detailed in the writing. Four of these 10 cases had pressure pneumothorax. All were in the correct side of the chest. Patients were basically females (10.7). The middle (range) age was 70.5 (89-55) a long time. Four patients required medical procedure (40%) and one patient, who was not worked on passed away (10%). Clinicians ought to know about this genuine intricacy. Unexplained chest agony, dyspnoea, and oxygen desaturation with stomach distension during ERCP must raise this chance. Early clinical acknowledgment and brief administration is basic to improve the result (20).

The following mechanisms have been proposed for post-ERCP pneumothorax and SCE: (i) after the interruption of the duodenal obstruction, air enters the retroperitoneal space; subsequently, air moves from the retroperitoneal space to the peritoneum, subcutaneous tissue, and mediastinum. Air spreads along the deep fascial planes (21). Deep fascia in the neck which is juxtaposed with diaphragmatic as well as esophageal hiatus and major airways of the thorax envelopes the esophagus and trachea. (ii) The air moves from the duodenum to the right anterior pararenal space after perforation. Thereafter, it flows to the posterior pararenal space to have access to the diaphragmatic hiatus which leads to cervical sister chromatid exchange (SCE), pneumothorax, or pneumomediastinum (iii). It is also assumed that air spreads along the perineural and perivascular sheaths to enter the mediastinum (iv). Alveolar rupture or porous diaphragm are considered two less feasible pathways due to Valsalva maneuver (18). 

The sort and seriousness of the leakage and clinical indications determine the treatment of post-ERCP perforation ought to be determined. Surgery has been recognized as the first-line treatment for type I and type II perforations. Nonetheless, the recent development of endoscopic treatments have opened the gates to treat perforation successfully with endoloop applications, endoscopic closure devices, and endoscopic clippings (13). 

Surgical intervention is not regarded as the first-line treatment for pneumothorax. The initial treatment includes broad-spectrum antibiotics, chest tube insertion, the administration of oxygen, and total parenteral nutrition. Nevertheless, the prognosis varies depending on the progress of the retroperitoneal perforation (22). However, it is worthy to note that surgical intervention is mandatory for patients with scope-induced perforation.

In conclusion from the data presented in the current study, it can be concluded that the utmost caution must be exercised by endoscopists while performing sphincterotomy, particularly in older female patients. As evidenced by the obtained results, most cases were effectively treated with nasogastric tube arrangement, antibiotics, nil orally, and chest tube drainage. Nonetheless, surgery was required in some patients. Moreover, it was found that a good prognosis can be expected in patients with ERCP-related pneumothorax, provided that the problem is diagnosed early.
